# Supplementary material for: Energy and biomass distribution in soil food webs of temperate and tropical forests
Source: Nat Commun. 2026 Jan 9;17:417. doi: 10.1038/s41467-025-68083-8 (PMC12796436; doi:10.1038/s41467-025-68083-8)
Supplement: Supplementary file 1 — Supplementary Information [file 41467_2025_68083_MOESM1_ESM.pdf]

Potapov A.M. et al. Energy and biomass distribution in soil food webs of temperate and tropical forests

Supplementary information

Supplementary Table 1. Sampling sites and their environmental characteristics across regions and forest types.

Site	Latitude	Longitude	Mean annual temperature (ºC)	Annual precipitation (cm)	pH	Litter_CN	NPP	Vegetation
**Monsoon forests (Vietnam)**								
Afzelia	11.4278	107.425583	26.0	219.9	5.5	47	817	*Lagerstroemia calyculata* dominates in canopy. *Mammea siamensis, Haldina cordifolia* and *Tetrameles nudiflora* are also abundant
Botanical garden	11.442067	107.432633	26.0	219.9	4.1	37.9	817	Dong Nai riverbank. Soil sandy. Polydominated forest, lots of large *Lagerstroemis calyculata* trees.
Dipterocarpus	11.44245	107.4025	26.0	219.9	5.0	37.8	817	Polydominated forest with young *Dipterocarpus* spp. trees
DST	11.42635	107.426033	26.0	219.9	6.3	33.6	817	*Dipterocarpus* sp. and *Ficus* sp. dominate
Reforestation	11.396017	107.37515	26.0	219.9	3.9	36.2	817	Planted in 1996-98, *Dipterocarpus* sp. dominates
River	11.44675	107.44055	26.0	219.9	4.2	40.2	817	Soil sandy. Dong Nai riverbank. *Dipterocarpus* spp. are dominating upper canopy. Abundant *Calamus* sp. in understory.
Tower	11.441233	107.401233	26.0	219.9	4.4	40.9	817	Old *Lagerstroemia calyculata* trees are dominating
Waterfall	11.411833	107.37835	26.0	219.9	4.1	38	817	*Lagerstroemia calyculata* dominates. *Tetrameles nudiflora* abundant
**Rainforests (Indonesia)**								
HF1	-2.15275	103.362	25.2	242.9	3.9	25.4	969	Mixed rainforest, Hutan Harapan national park, loam Acrisol
HF2	-2.158166667	103.33375	25.2	242.9	3.8	28.9	969	Mixed rainforest, Hutan Harapan national park, loam Acrisol

HF3	-2.175027778	103.3327222	25.2	253.9	4.0	27.6	969	Mixed rainforest, Hutan Harapan national park, loam Acrisol
HF4	-2.187555556	103.3426111	25.2	243.7	4.1	33.9	969	Mixed rainforest, Hutan Harapan national park, loam Acrisol
BF1	-1.995138889	102.75225	25.2	277.3	3.9	25.6	969	Mixed rainforest, Bukit Duabelas national park, clay Acrisol
BF2	-1.981972222	102.75075	25.2	277.3	4.0	28	969	Mixed rainforest, Bukit Duabelas national park, clay Acrisol
BF3	-1.94275	102.5813056	25.2	286.6	3.2	35.8	969	Mixed rainforest, Bukit Duabelas national park, clay Acrisol
BF4	-1.941944444	102.5806389	25.2	286.6	3.4	34.3	969	Mixed rainforest, Bukit Duabelas national park, clay Acrisol
**Beech forests (Germany)**								
HEW6	51.26772	10.239378	7.5	77.8	4.3	36.8	633	Beech forest (*Fagus sylvatica*) with srong wood (c.70 years old)
HEW21	51.194278	10.319005	7.5	77.8	4.9	30.8	633	Beech forest (*Fagus sylvatica*) with srong wood (c.70 years old)
HEW22	51.33715	10.359279	7.5	76.1	5.5	33.6	633	Beech forest (*Fagus sylvatica*) with srong wood (c.70 years old)
HEW47	51.178935	10.378344	7.5	69.5	4.4	32.4	633	Beech forest (*Fagus sylvatica*) with srong wood (c.70 years old)
SEW5	53.057034	13.885366	7.5	56	3.1	31.5	633	Beech forest (*Fagus sylvatica*) with srong wood (c.70 years old)
SEW35	52.911268	13.853419	7.5	55.4	3.4	27.2	633	Beech forest (*Fagus sylvatica*) with srong wood (c.70 years old)
SEW36	52.951418	13.754141	7.5	56.2	3.4	31.7	633	Beech forest (*Fagus sylvatica*) with srong wood (c.70 years old)
SEW37	52.940022	13.782612	7.5	56.2	3.3	31.6	633	Beech forest (*Fagus sylvatica*) with srong wood (c.70 years old)

**Mixed broadleaved forests (European Russia)**								
BL9c	52.594845	38.923564	7.1	56.7	6.2	28	418	Broadleaved forest (*Quercus robur*, *Betula pendula, Prunus padus*), no wildfire, Chernozem chernic
BL10f	52.594845	38.923564	7.1	56.7	5.1	33.3	418	Broadleaved forest (*Prunus padus, Aegopodium podagraria, Chelidonium majus*), after wildfire in 2010, Chernozem chernic
BL11c	52.580094	39.000751	7.1	57	5.4	22.3	418	Broadleaved forest (*Quercus robur, Prunus padus*), no wildfire, Phaeozem albic
BL12f	52.580094	39.000751	7.1	57	5.2	28	418	Broadleaved forest (with *Ribes nigrum*), after wildfire in 2010, Phaeozem albic
**Southern taiga forests (European Russia)**								
ST21c	56.155257	32.817537	5.2	63.8	5.5	28.6	552	Spruce forest (*Picea abies, Sorbus aucuparia*), no wildfire, Albeluvisol umbric
ST22f	56.155257	32.817537	5.2	63.8	5.9	22.8	552	Spruce forest (*Picea abies, Rubus idaeus, Sorbus aucuparia*), after wildfire in 2010, Albeluvisol umbric
ST23c	56.140112	32.718251	5.2	63.8	5.1	31.2	552	Spruce forest (*Picea abies, Betula pendula*), no wildfire, Albeluvisol umbric
ST24f	56.140112	32.718251	5.2	63.8	5.2	30.9	552	Spruce forest (*Populus tremula, Picea abies*), after wildfire in 2010, Albeluvisol umbric

Supplementary Table 2. Effect of climate type (tropical vs temperate) on the trophic functions in forest soil food webs (proportions of the total). Results of beta regressions run for each function separately. Two-sided Wald chi-square test results are shown; degree of freedom is one.

**Trophic function**	**Chi-squared**	**p**
Predation	18.6	1.6×10^{-5}
Algivory	1.2	2.7×10^{-1}
Herbivory	51.9	5.9×10^{-13}
Bacterivory	34.7	3.9×10^{-9}
Fungivory	10.5	1.2×10^{-3}
Litter consumption	13.8	2.0×10^{-4}
Wood consumption	2.0	1.6×10^{-1}
Soil consumption	1.2	2.7×10^{-1}

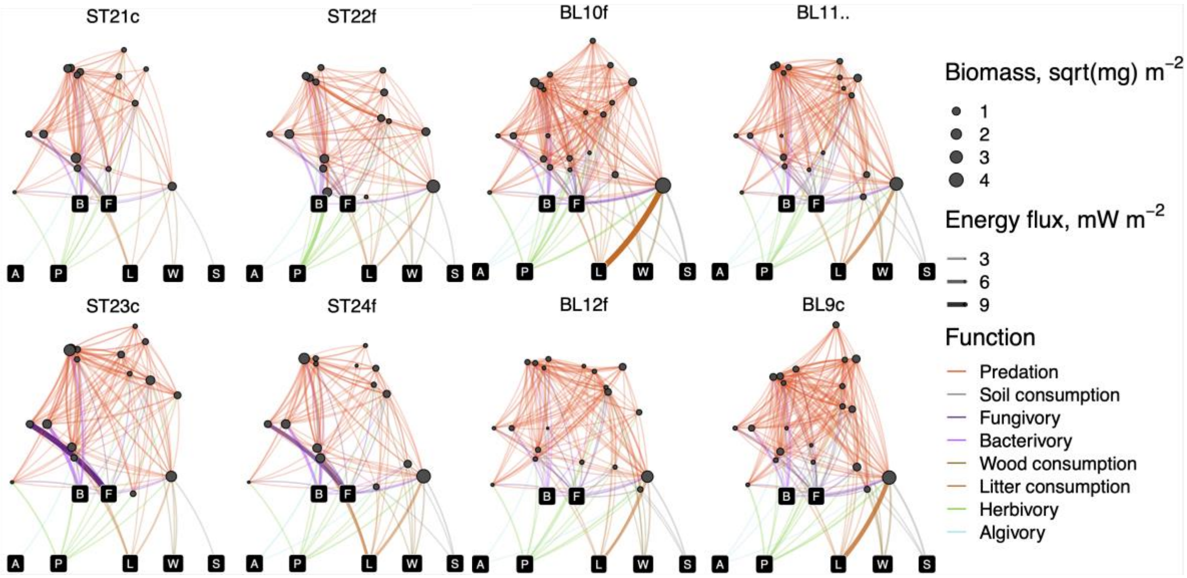

Supplementary Figure 1. Reconstructed soil food webs in southern taiga (ST) and mixed broadleaved (BL) forests. Animal nodes are shown with points, resource nodes are shown with black labelled rectangles. Size of nodes corresponds to the biomasses, thickness and brightness of lines corresponds to the energy fluxes. Colours group energy fluxes by resource-based trophic functions and predation.

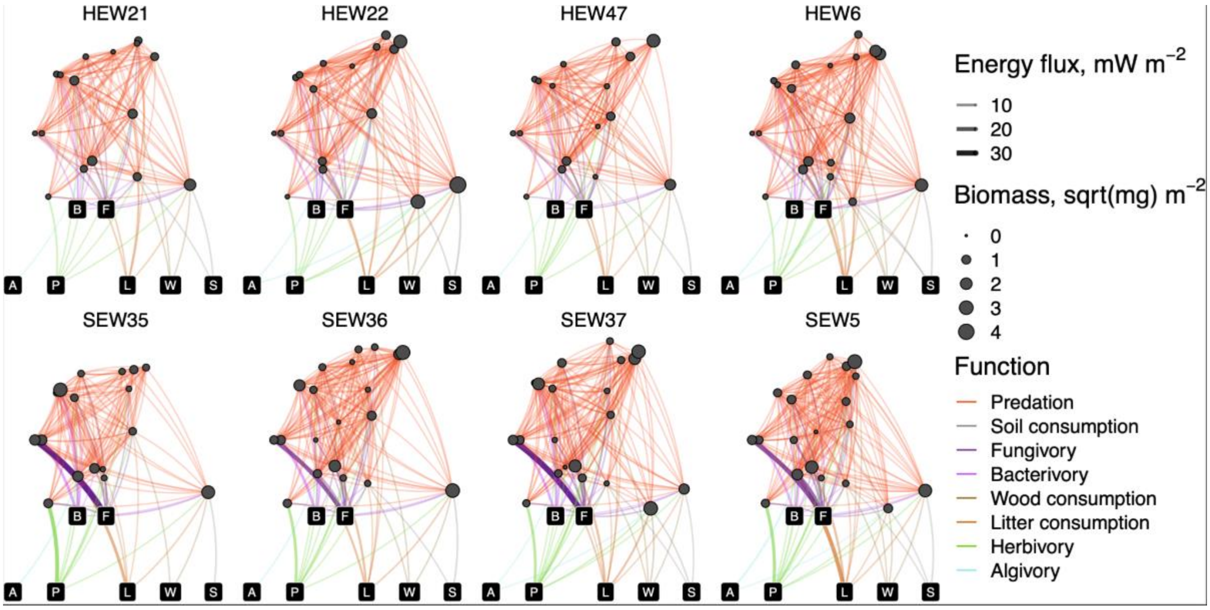

Supplementary Figure 2. Reconstructed soil food webs in beech forests. Animal nodes are shown with points, resource nodes are shown with black labelled rectangles. Size of nodes corresponds to the biomasses, thickness and brightness of lines corresponds to the energy fluxes. Colours group energy fluxes by resource-based trophic functions and predation. Each network represents one sampling site.

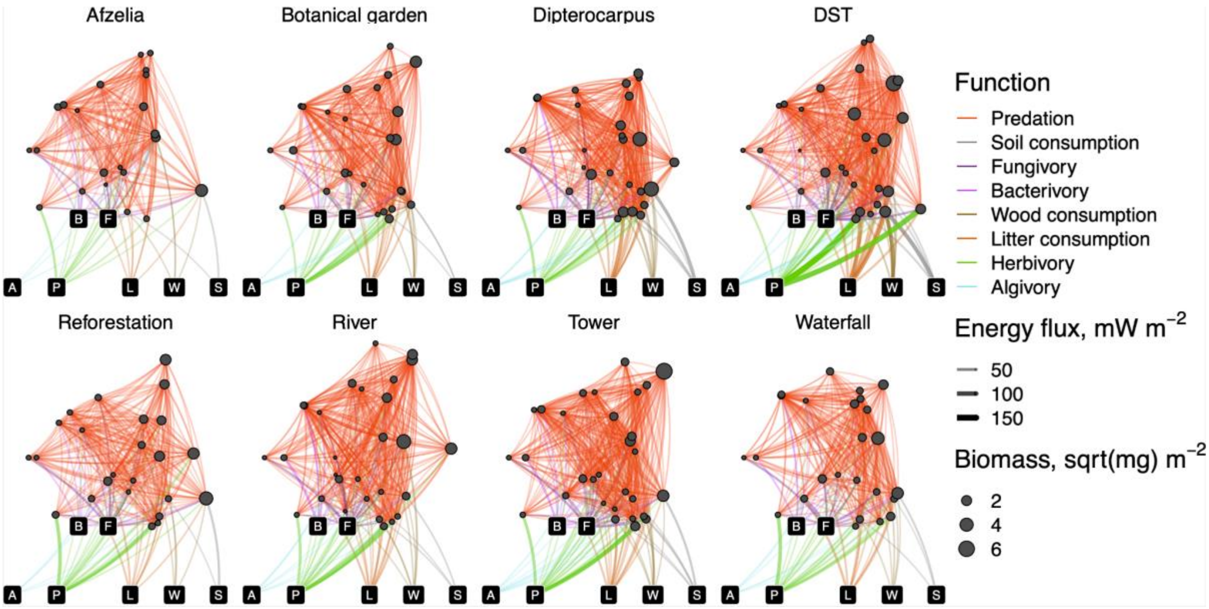

Supplementary Figure 3. Reconstructed soil food webs in monsoon forests. Animal nodes are shown with points, resource nodes are shown with black labelled rectangles. Size of nodes corresponds to the biomasses, thickness and brightness of lines corresponds to the energy fluxes. Colours group energy fluxes by resource-based trophic functions and predation. Each network represents one sampling site.

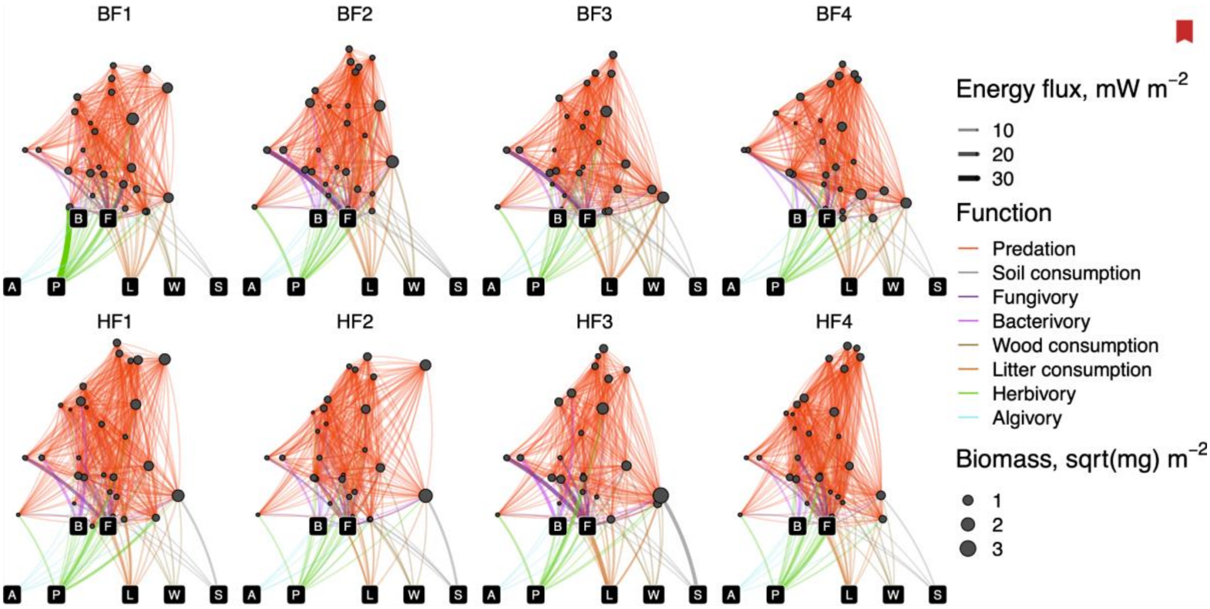

Supplementary Figure 4. Reconstructed soil food webs in rainforests. Animal nodes are shown with points, resource nodes are shown with black labelled rectangles. Size of nodes corresponds to the biomasses, thickness and brightness of lines corresponds to the energy fluxes. Colours group energy fluxes by resource-based trophic functions and predation. Each network represents one sampling site.

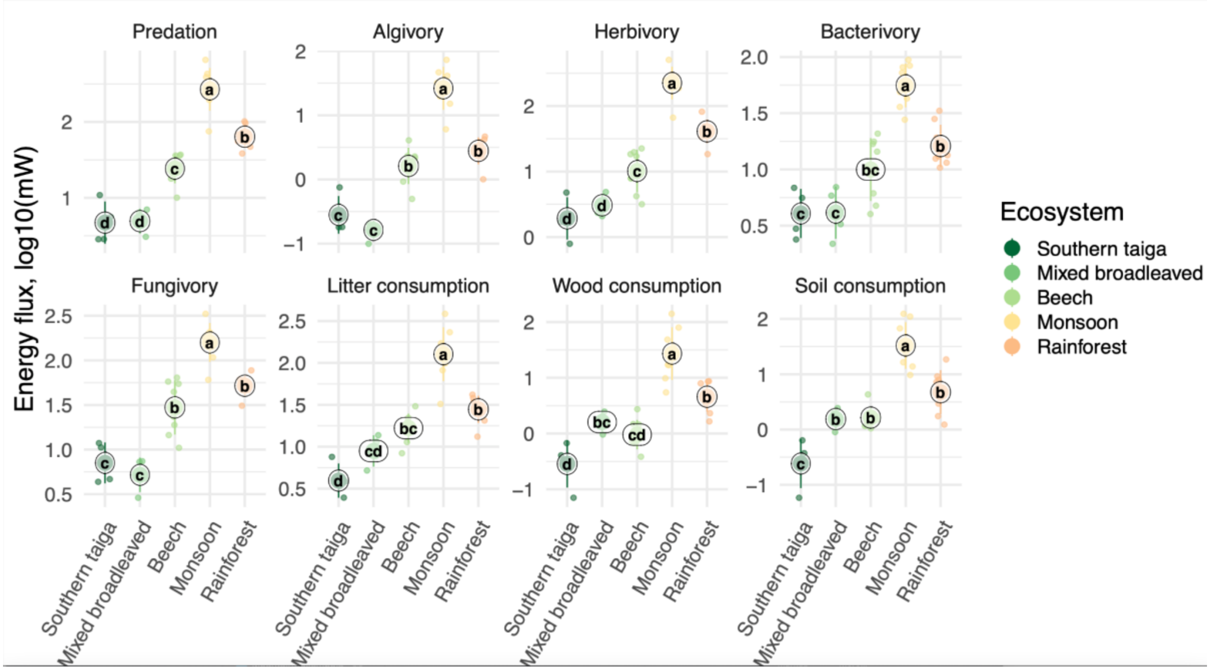

Supplementary Figure 5. Trophic functions in soil food webs in forests of different climatic regions. Each trophic function represents a sum of all energy fluxes outgoing from a specific resource/prey: predation – animal nodes, algivory – algae, herbivory – living plants, bacterivory – bacteria, fungivory – fungi, litter consumption – litter, wood consumption – dead wood, soil consumption – soil organic matter. Means ± 1 SD are shown, each point represents a site. Means sharing the same letter within forest types are not significantly different (Tukey HSD test for beta regressions; n = 4 for taiga and broadleaved forests and 8 for other). Please, note that absolute fluxes (in mW) are shown in this graph.

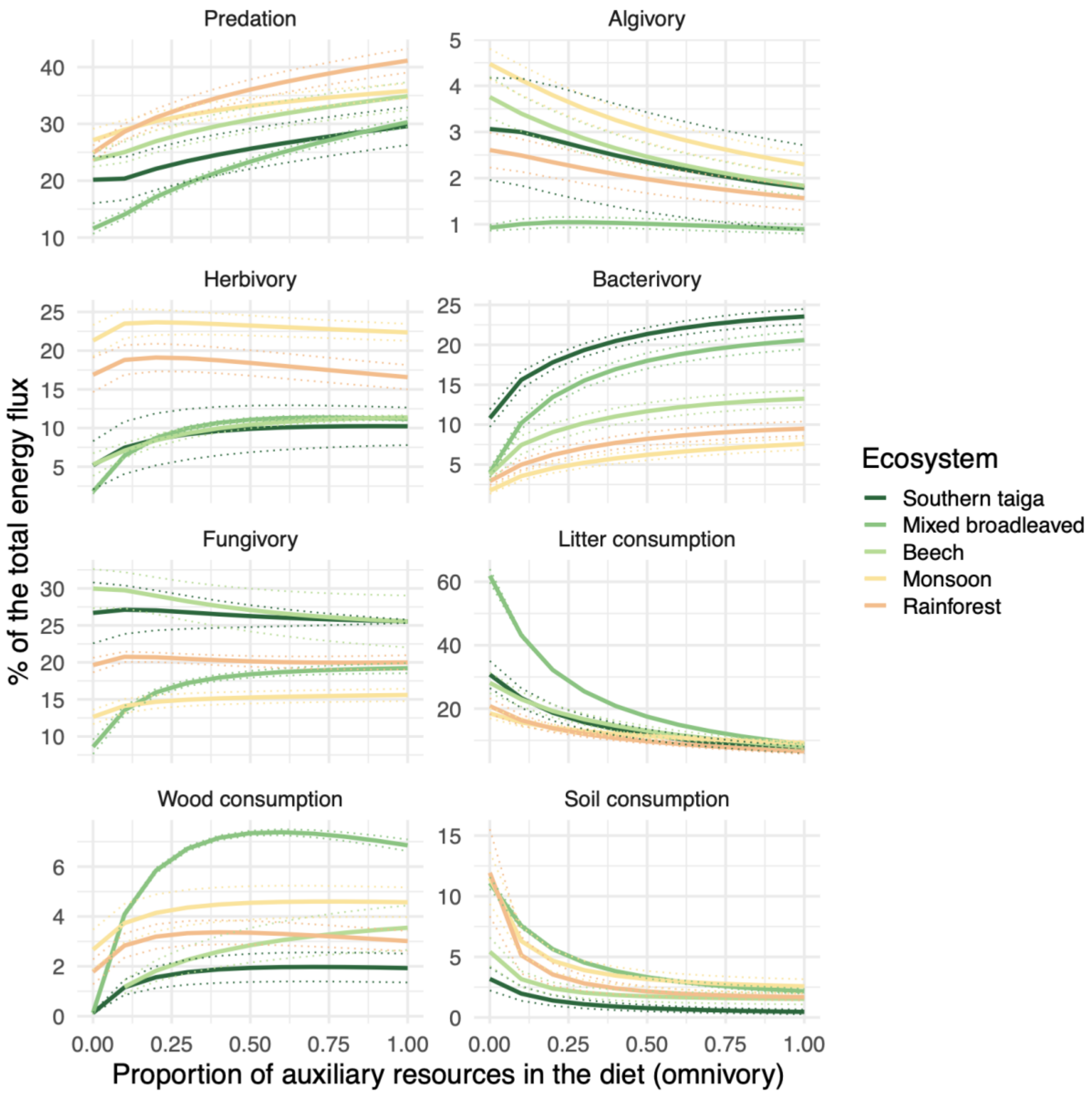

Supplementary Figure 6. Sensitivity analysis of the food-web reconstruction. Changes in the percentage of different trophic functions in the total energy flux (mean value) against the omnivory coefficient is plotted with solid lines. Dotted lines represent standard errors (n = 4 for taiga and broadleaved forests and 8 for other). Colours show different forest types.
