# Supplementary material for: Energy and biomass distribution in soil food webs of temperate and tropical forests
Source: Nat Commun. 2026 Jan 9;17:417. doi: 10.1038/s41467-025-68083-8 (PMC12796436; doi:10.1038/s41467-025-68083-8)
Supplement: Supplementary file 2 — Description of Additional Supplementary Information [file 41467_2025_68083_MOESM2_ESM.pdf]

SoilFoodWeb - Raw data.csv = main raw data filecontaining data on body mass, density and biomass persquare meter of taxonomic group of soil invertebratesfrom all sites.

Raw_metabolicRates.csv = coefficients used formetabolic rate calculations, compiled from Ehnes et al.201121, Brown et al. 200414 and Klekowski et al. 1972.Klekowski, R. Z.; Wasilewska, L.; Paplinska, E. OxygenConsumption By Soil-Inhabiting Nematodes.Nematologica 1972, 18 (3), 391–403.https://doi.org/10.1163/187529272X00665.

Raw_isotopes.xlsx = stable isotope composition of Cand N in soil invertebrates compiled from papers onGerman (Klarner et al. 201466), Russian (Potapov et al.202134), Indonesian (Zhou et al. 202446) andVietnamese forests (Tsurikov et al. 2019). Tsurikov, S.M.; Ermilov, S. G.; Tiunov, A. V. Trophic Structure of aTropical Soil- and Litter-Dwelling Oribatid MiteCommunity and Consistency of Trophic Niches acrossBiomes. Exp Appl Acarol 2019, 78, 29–48.https://doi.org/10.1007/s10493-019-00374-4.

Raw_traits.xlsx = Feeding preferences, protection traitsand vertical stratification of soil invertebrate taxa andtheir guild assignment. The table is based on Potapov202216.

Site_descriptions.xlsx = Geographical coordinates,descriptions and environmental factors for all samplingsites.

SoilFoodWeb - R script publication.R = Full annotatestatistical code to produce descriptive statistics,reconstruct food webs, calculate energy fluxes, dostatistical analyses and produce figures.
