# Supplementary material for: Energy and biomass distribution in soil food webs of temperate and tropical forests
Source: Nat Commun. 2026 Jan 9;17:417. doi: 10.1038/s41467-025-68083-8 (PMC12796436; doi:10.1038/s41467-025-68083-8)
Supplement: Supplementary file 4 — Reporting Summary [file 41467_2025_68083_MOESM4_ESM.pdf]

nature portfolio

Corresponding author(s): Anton Potapov

Last updated by author(s): 05/09/2024

Reporting Summary

Nature Portfolio wishes to improve the reproducibility of the work that we publish. This form provides structure for consistency and transparency in reporting. For further information on Nature Portfolio policies, see our Editorial Policies and the Editorial Policy Checklist.

Statistics

For all statistical analyses, confirm that the following items are present in the figure legend, table legend, main text, or Methods section.

n/a	Confirmed	
☐	☒	The exact sample size (*n*) for each experimental group/condition, given as a discrete number and unit of measurement
☐	☒	A statement on whether measurements were taken from distinct samples or whether the same sample was measured repeatedly
☐	☒	The statistical test(s) used AND whether they are one- or two-sided<br>*Only common tests should be described solely by name; describe more complex techniques in the Methods section.*
☐	☒	A description of all covariates tested
☐	☒	A description of any assumptions or corrections, such as tests of normality and adjustment for multiple comparisons
☐	☒	A full description of the statistical parameters including central tendency (e.g. means) or other basic estimates (e.g. regression coefficient) AND variation (e.g. standard deviation) or associated estimates of uncertainty (e.g. confidence intervals)
☐	☒	For null hypothesis testing, the test statistic (e.g. *F*, *t*, *r*) with confidence intervals, effect sizes, degrees of freedom and *P* value noted<br>*Give P values as exact values whenever suitable.*
☒	☐	For Bayesian analysis, information on the choice of priors and Markov chain Monte Carlo settings
☒	☐	For hierarchical and complex designs, identification of the appropriate level for tests and full reporting of outcomes
☒	☐	Estimates of effect sizes (e.g. Cohen's *d*, Pearson's *r*), indicating how they were calculated

Our web collection on statistics for biologists contains articles on many of the points above.

Software and code

Policy information about availability of computer code

Data collection	No software was used during the data collection.
Data analysis	Data analysis was implemented in R v4.2.0 with R studio interface v2023.06.2+561 (RStudio, PBC). The following packages were used: lme4 v1.1-33, tidyverse v2.0.0, plyr v1.8.8, reshape v0.8.9, reshape2 v1.4.4, fluxweb v0.2.0, food-web reconstruction code and code used for statistical analyses are available from the paper Supplementary Data 1 and Figshare https://doi.org/10.6084/m9.figshare.29341058

For manuscripts utilizing custom algorithms or software that are central to the research but not yet described in published literature, software must be made available to editors and reviewers. We strongly encourage code deposition in a community repository (e.g. GitHub). See the Nature Portfolio guidelines for submitting code & software for further information.

Data

Policy information about availability of data

All manuscripts must include a data availability statement. This statement should provide the following information, where applicable:

- Accession codes, unique identifiers, or web links for publicly available datasets
- A description of any restrictions on data availability
- For clinical datasets or third party data, please ensure that the statement adheres to our policy

Raw data underlying results of the present paper are available from Supplementary Data 1.

Research involving human participants, their data, or biological material

Policy information about studies with human participants or human data. See also policy information about sex, gender (identity/presentation), and sexual orientation and race, ethnicity and racism.

Reporting on sex and gender	N/A
Reporting on race, ethnicity, or other socially relevant groupings	N/A
Population characteristics	N/A
Recruitment	N/A
Ethics oversight	N/A

Note that full information on the approval of the study protocol must also be provided in the manuscript.

Field-specific reporting

Please select the one below that is the best fit for your research. If you are not sure, read the appropriate sections before making your selection.

☐ Life sciences ☐ Behavioural & social sciences ☒ Ecological, evolutionary & environmental sciences

For a reference copy of the document with all sections, see nature.com/documents/nr-reporting-summary-flat.pdf

Ecological, evolutionary & environmental sciences study design

All studies must disclose on these points even when the disclosure is negative.

Study description	The study compares different forest types (southern taiga, mixed broadlevaed, beech, monsoon and rainforests), each represented by 4 or 8 sites (=replicates). The total sample size is 32 sites.
Research sample	The study analyses invertebrates extracted from soil and litter including nematodes, arthropods and earthworms
Sampling strategy	Each group (nematodes, mesofauna, macrofauna) was sampled with targeted sampling method. From 3 to 18 samples were taken per site to account for spatial heterogeneity. The samples were averaged and the statistical n was 32 sites.
Data collection	Data were collected using manual counting, measuring and identification of invertebrates under the microscope
Timing and spatial scale	Sampling was done from 2013 to 2021 depending on the site in the peak vegetation activity periods. The sties in Indonesia were assessed in two years (2013 and 2016) and the results were averaged. Each site measured ca. 50 x 50 meters
Data exclusions	N/A
Reproducibility	Each site was represented by multiple samples averaged (3-18). The sties in Indonesia were assessed in two years and the results were averaged.
Randomization	Samples within sites were positioned randomly; site selection followed representation of the dominant forest type(s) and was also random
Blinding	N/A

Did the study involve field work? ☐ Yes ☐ No

Field work, collection and transport

Field conditions	The data were collected in 4 different regions, spanning from temperate (Germany, European Russia) to tropical (Vietnam, Indonesia) climates
Location	Germany; European Russia (Nelidovo, Kaluga); Vietnam; Indonesia (Sumatra)
Access & import/export	The permits for collection and export of the samples were granted by corresponding authorities. For Indonesia - the Indonesian Ministry of Forestry (PHKA), Directorate General of Nature Resources and Ecosystem Conservation (KSDAE), and the Indonesian Institute of Sciences (LIPI). Collection permit no. S.07/KKH-2/2013 issued by PHKA, and export permit by LIPI (register file no. 24/SI/MZB/IV/2014) and PHKA (no. 125/KKH-5/TRP/2014).
Disturbance	Work on the study sites was implemented with care, to minimize disturbance. Whenever possible, manipulations with samples were

done in a laboratory, outside the field sampling areas.

Reporting for specific materials, systems and methods

We require information from authors about some types of materials, experimental systems and methods used in many studies. Here, indicate whether each material, system or method listed is relevant to your study. If you are not sure if a list item applies to your research, read the appropriate section before selecting a response.

Materials & experimental systems

n/a	Involved in the study
☒	☐ Antibodies
☒	☐ Eukaryotic cell lines
☒	☐ Palaeontology and archaeology
☐	☒ Animals and other organisms
☒	☐ Clinical data
☒	☐ Dual use research of concern
☒	☐ Plants

Methods

n/a	Involved in the study
☒	☐ ChIP-seq
☒	☐ Flow cytometry
☒	☐ MRI-based neuroimaging

Animals and other research organisms

Policy information about studies involving animals; ARRIVE guidelines recommended for reporting animal research, and Sex and Gender in Research

Laboratory animals	N/A
Wild animals	Only invertebrate animals (nematodes, arthropods and earthworms) were collected and killed using ethanol during the study. This was necessary to assess biomass and community composition. We assessed soil arthropod and earthworm communities using extractors. Presumably several hundreds of species (mainly unidentified) were collected without selection for sex, age or strains.
Reporting on sex	All invertebrate animals were collected without selection for sex. No data on sex ratios are available.
Field-collected samples	Collected soil samples were transported to the lab within 2-3 days for extraction and laboratory analyses. All invertebrates were stored in 75-95% ethanol solution under room temperature.
Ethics oversight	Ethical approval was not required for the study.

Note that full information on the approval of the study protocol must also be provided in the manuscript.

Plants

Seed stocks	N/A
Novel plant genotypes	N/A
Authentication	N/A
